# CT imaging in post-resuscitation care of non-traumatic resuscitation room patients in German hospitals

**DOI:** 10.1186/s12873-025-01216-w

**Published:** 2025-04-15

**Authors:** Akvile Juskeviciute, Milda Aleknonyte Resch, Bernhard Kumle, Hans Jörg Busch, Uwe Janssens, Guido Michels, Lars Roman Herda, Martin Faber, Sabine Merz, Michael Reindl, Christoph Wasser, Stefan Kornstaedt, Patrick Langguth, Kevin Schulte, Michael Bernhard, Martin Pin, Domagoj Schunk

**Affiliations:** 1 Department of Intensive Care Medicine, University Medical Center Hamburg-Eppendorf (UKE), Hamburg, Germany; 2 Department of Business Informatics (Process Analytics), Christian-Albrechts-University of Kiel, Kiel, Germany; 3 Emergency Department, Black Forest-Baar Hospital, Villingen Schwenningen, Germany; 4 Department of Emergency Medicine, Faculty of Medicine, University Hospital, University of Freiburg, Freiburg, Germany; 5 Department of Internal Medicine and Internal Intensive Care, Gastroenterology, Cardiology and Nephrology, St.-Antonius Hospital, Eschweiler, Germany; 6 Emergency Department Center, Hospital Barmherzige Brüder Trier, Trier, Germany; 7 Emergency Department, Medical Faculty, University Hospital of Duesseldorf, Heinrich-Heine University, Duesseldorf, Germany; 8 Emergency Department, Florence-Nightingale Hospital Duesseldorf, Duesseldorf, Germany; 9 Interdisciplinary Emergency Department, University Hospital Schleswig-Holstein (UKSH), Campus Kiel, Germany; 10 Department of Nephrology and Hypertension, University Hospital Schleswig-Holstein (UKSH), Campus Kiel, Germany; 11 Department of Radiology and Neuroradiology, University Hospital Schleswig-Holstein (UKSH), Campus Kiel, Germany; 12 Emergency Department, Hospital Osnabrück, Osnabrück, Germany; 13 Department of Cardiology, Staedtisches Klinikum Braunschweig, Brunswick, Germany; 14 Emergency Department, Hospital Ameos St. Clemens Oberhausen, Oberhausen, Germany; 15 Emergency Department, Hospital Robert Bosch Stuttgart, Stuttgart, Germany; 16 Emergency Department, Hospital Ingolstadt, Ingolstadt, Germany; 17 German Society for Interdisciplinary Emergency and Acute Medicine (DGINA), Berlin, Germany; 18 German Society of Medical Intensive Care and Emergency Medicine (DGIIN), Berlin, Germany; 19 German Interdisciplinary Association for Intensive Care and Emergency Medicine (DIVI), Berlin, Germany; 20 German Society of Cardiology (DGK), Duesseldorf, Germany

**Keywords:** Out-of-hospital cardiac arrest (OHCA), Whole-body computed tomography, Cardiac-arrest-center (CAC), Germany, Emergency department, Resuscitation room, Postresuscitation protocol

## Abstract

**Background:**

The procedures and locations where patients are admitted to hospitals and subsequently diagnosed after out-of-hospital cardiac arrest (OHCA) in Germany exhibit considerable heterogeneity. Specifically, advanced imaging diagnostic methods via computed tomography (CT) show significant variation in both timing and execution. However, echocardiography (ECHO) is not an alternative to CT in this setting, as both modalities serve distinct diagnostic purposes. This study aimed to comprehensively analyze the status quo analysis of current procedures in German emergency departments (EDs) regarding early-phase (within the first six hours) CT imaging diagnostics after resuscitation and the treatment of critically ill patients in the ED resuscitation room.

**Methods:**

An anonymized cross-sectional study was conducted from November 28, 2023, to February 18, 2024, using an online survey platform (https://www.surveymonkey.de) with a standardized questionnaire. The survey targeted 994 medical directors of German EDs and was distributed through the mailing lists of the German Society for Interdisciplinary Emergency and Acute Medicine (DGINA) and the German Interdisciplinary Association for Intensive Care and Emergency Medicine (DIVI). The Medical Faculty of Christian-Albrechts-University Kiel granted ethical approval (D 586/22). An expert panel reviewed the questionnaire to ensure validity and minimize bias. All statistical analyses, including both descriptive and inferential statistics, were conducted using R software.

**Results:**

Out of 994 hospitals contacted, 182 hospitals from 15 German federal states participated, yielding a response rate of 18.3%. The overall completion rate for the whole questionnaire was 12.2% (*n* = 121/994). In the survey, 9.6% (*n* = 15/157) of hospitals reported having CT within the resuscitation room, while 70.1% (*n* = 119/157) had CT within a range of 50 m of the resuscitation room. A standard operating procedure (SOP)/postresuscitation protocol for patients suffering from OHCA was available for 61.1% (*n* = 88 yes, *n* = 56 no) of the hospitals. A specific postresuscitation CT protocol (postrCT protocol) was used by 30.0% (*n* = 48 yes, *n* = 93 no) of the hospitals, with 59.2% (*n* = 29) receiving a head-to-pelvis CT (whole-body CT). In hospitals without a CT protocol (*n* = 84), echocardiography (82.1%, *n* = 69), abdominal ultrasound (61.9%, *n* = 52), and non-contrast CT of the head (47.6%, *n* = 40) are used for distinctive diagnostics. Cardiac Arrest Center (CAC)-certified hospitals were significantly more likely to have a SOP/postresuscitation protocol (91.9 vs. 49.0%, *p* < 0.001) and a specific postrCT protocol (63.2 vs. 22.1%, *p* < 0.001) than noncertified hospitals.

**Conclusion:**

Currently, there is no nationwide standardized protocol for imaging diagnosis in patients after OHCA in German EDs. Protocols are more often used in CAC hospitals in Germany then in non-certified hospitals. Given the limitations of survey-based research, results should be interpreted with caution regarding their representativeness across all German EDs and further prospective studies including mortality and neurological outcomes are warranted.

**Supplementary Information:**

The online version contains supplementary material available at 10.1186/s12873-025-01216-w.

## Introduction

In Germany, with a population of approximately 83 million people, approximately 55,000 patients with cardiac arrest were resuscitated by emergency medical services (EMS) in 2023. Increased public awareness and structural improvements in out-of-hospital medical care have led to favourable developments. From 2022 to 2023, the proportion of telephone-assisted resuscitations and the number of CPRs performed by first responders increased significantly, and the number of ambulance arrivals improved. Through targeted out-of-hospital measures, 41.4% of patients achieved a return of spontaneous circulation (ROSC) after out-of-hospital cardiac arrest (OHCA) in 2023. However, survival to hospital discharge will remain low at 10.4% in 2023 [1, 2].

Since 2018 Cardiac Arrest Centers (CAC) in Germany are specialized hospitals certified to provide comprehensive post-resuscitation care for patients who have suffered out-of-hospital cardiac arrest (OHCA). These centers are equipped with advanced diagnostic and therapeutic capabilities, including 24/7 cardiac catheterization labs, targeted temperature management, and multimodal neurological monitoring, ensuring optimal treatment and improved patient outcome [3].

Standardized diagnostic tests, particularly computed tomography (CT) scans, play a significant role in the early stages of treatment. However, the majority of resuscitated patients after a nontraumatic OHCA do not receive standardized CT scans in terms of scope or timing [4–6]. Although there is no consensus yet, current European resuscitation guidelines recommend considering a head CT and/or a pulmonary artery CT (CT-PA) only if the causes of cardiac arrest are unclear [7]. In practice, patients who have suffered cardiac arrest and a history of traumatic events are immediately subjected to whole-body CT. However, 96.9% of resuscitation cases are classified as nontraumatic by emergency physicians or paramedics [1].

A study by Branch et al. [4] showed that performing a head-to-pelvis CT within the first 6 h in patients with unclear OHCA significantly increased the identification of the cause of cardiac arrest (92 vs. 72%) and reduced the delay (> 6 h) in identifying critical diagnoses by 81% [4]. In addition, early CT is crucial for the rapid and efficient detection of potential resuscitation complications and unclear causes of OHCA [4–12]. Zotsmann et al. (2020) performed whole-body CTs within 24 h of sudden cardiac arrest in patients with eCPR and identified the cause of sudden cardiac arrest in 16.5% of patients, with 19.4% of the findings being severe enough to terminate therapy [13].

To date, the factors influencing the decision to undergo a postresuscitation CT in patients with OHCA in Germany have not been identified. Given the lack of data, this study aimed to collect information on emergency department (ED) processes in the first hours after admission in nontraumatic OHCA survivors. In addition, the study compared differences in care between CAC-certified hospitals and non-CAC-certified hospitals.

## Materials and methods

This was an anonymized cross-sectional study conducted using an online survey and a standardized questionnaire. The online survey was approved by the Ethics Committee of the Medical Faculty of Christian-Albrechts-University of Kiel, Germany (Az D586/22).

The questionnaire was developed by a group of experts representing professional societies based on current resuscitation guidelines and CAC certification guidelines [7, 14]. To ensure clarity, validity, and reliability of the questionnaire, a pretest was conducted with a panel of emergency medicine and radiology experts from the participating professional societies before distribution. Additionally, the questionnaire was optimized in four online consensus rounds and revised based on pretest feedback.

The editorial work was carried out by representatives of the working group “Resuscitation Room - Post-Resuscitation Imaging” of the German Society for Interdisciplinary Emergency and Acute Medicine (DGINA) in collaboration with the German Interdisciplinary Association for Intensive Care and Emergency Medicine (DIVI), the German Society for Medical Intensive Care and Emergency Medicine (DGIIN), and the German Society of Cardiology (DGK).

The questionnaire consists of 77 questions covering the whole spectrum regarding the process of diagnostic pathways and SOP’s used for patients after OHCA, as well as the hospital structure. After providing informed consent (in accordance with the German National General Data Protection Regulation), participants were asked about their relationship with pediatric EDs. A sole leading position in a pediatric ED was an exclusion criterion, as the survey focused on patients aged 18 years and older.

The survey was created and formatted using the online survey platform SurveyMonkey^®^ (https://www.surveymonkey.de). Participants were medical directors of German EDs who received a link to the online questionnaire via the mailing lists of the German Society for Interdisciplinary Emergency and Acute Medicine e.V. (DGINA) and the German Interdisciplinary Association for Intensive Care and Emergency Medicine (DIVI). The survey was conducted over a 12-week period, with reminder emails sent every four weeks.

### Statistical analysis

Duplicate checks were performed to ensure data validity and reliability, followed by data analysis using R software (v. 4.4.1). Plots were generated using the ggplot2 package, Sankey MATIC or DataGraph 5.3. (Visual Data Tools, Inc. 2006–2024). The analyses in this study were carried out in two steps. The first step was descriptive statistics were absolute frequencies and percentages were analyzed. In the second step, differences between CACs and non-CAC-certified hospitals were tested using the chi-square test with Yates correction and Fisher’s exact test due to unequal group sizes. As there were a total of seven statistical tests, the significance level was adjusted using the Bonferroni correction, resulting in a significance level of 0.05/7 = 0.0071.

## Results

**Fig. 1:**
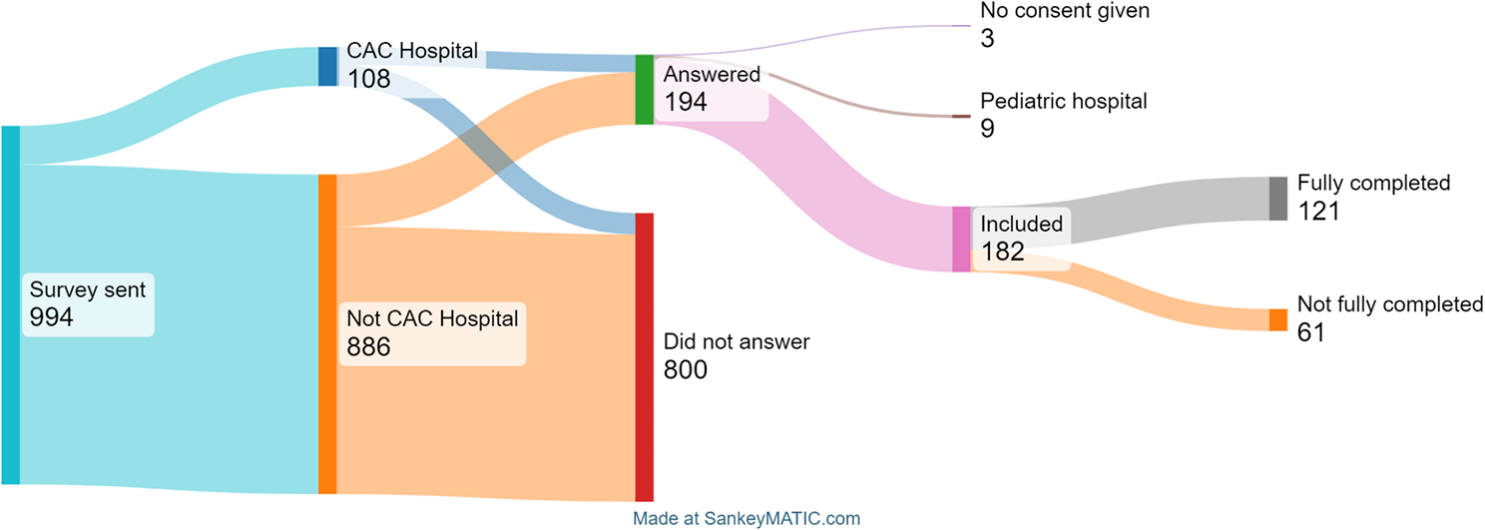
Flow diagram depicting the number of hospital EDs included and the number of surveys completed all questions “fully completed” (grey bar) vs. hospitals that participated but did not answer all questions “Not fully completed”

The survey was sent to 994 hospitals and their medical directors of German EDs, ensuring expertise in emergency care decision-making, including 108 CAC-certified hospitals. A total of 194 hospitals participated, but 3 declined to consent, and 9 solely pediatric EDs were excluded. The final study sample consisted of 182 hospitals (Fig. 1) yielding an overall completion rate for all questions in the questionnaire with 12.2% (121/994).

### Structure and environment

The distribution of ED structures according to the § 136 SGB V (nationwide German requirements for participation in emergency medicine) among the respondents representing 15 out of 16 German federal states was as follows: 31.7% (*n* = 53) in hospitals with basic emergency care, 33.5% (*n* = 56) in facilities with extended emergency care, and 34.7% (*n* = 58) in hospitals with comprehensive emergency care. Almost all the hospitals were organized as interdisciplinary EDs (97.7%, *n* = 166).

In addition, 66.2% (*n* = 104) of the respondents reported that a radiologist was available 24 h a day in the ED. In 73.0% (*n* = 111) of the patients, 24/7 had access to teleradiology therapy. Furthermore, 85.4% (*n* = 143) of the surveyed hospitals reported having a Medical Technical Radiology Assistant (MTRA) available 24/7 during resuscitation room treatment. The cardiac catheterization laboratory (CCL) is usually located on a different floor from the ED (35.4%, *n* = 51), while the second-largest group had the CCL in close proximity (less than 50 m) (33.3%, *n* = 48).

### Process and timepoint of CT examination

More than half of the ED medical directors (52.9%, *n* = 81) reported performing a CT examination within 6 h after a CCL examination for OHCA patients and after a successful intervention (e.g., in the case of a heart attack). In addition, 65.7% (*n* = 99) of the patients who underwent a CCL examination without intervention underwent a CT scan, and the cause of OHCA was not clearly determined. The transfer locations for patients after non traumatological OHCA did not change in 59.7% (*n* = 86 out of *n* = 144) of the hospitals, depending on the suspected etiology. In OHCA cases with suspected acute coronary syndrome, 85.5% (*n* = 106 out of *n* = 124) of the hospitals transfer their patients directly to the CCL, furthermore the CLL as the primary location in cardiac cause is in 32.3% (*n* = 40) an ongoing resuscitation if ROSC is not achieved, and 29.8% (*n* = 37) hospitals declare to use the CLL pathway if anamnestic indications of a cardiac event are obvious (e.g., the patient previously clutched their chest, sudden cardiac death during sports, or a known history of coronary artery disease (CAD) or heart failure). In cases of suspected noncardiac causes, 77.1% (*n* = 111 out of *n* = 144) of the patients were transferred to the ED, and 16.7% (*n* = 24 out of *n* = 144) were transferred to the intensive care unit (ICU).

### Postresuscitation CT protocol

Currently, only 30.0% (*n* = 48) of the 93 German EDs answering this question have a CT postresuscitation protocol. Almost all hospitals with a CT postresuscitation protocol (96.8%; *n* = 46) had an interdisciplinary central ED. However, a resuscitation room is usually not present in the ICU (72.3%, *n* = 34). In 70.1% (*n* = 33) of the EDs, a CT scanner was placed within 50 m of the resuscitation room. In 19.2% (*n* = 9) of the EDs, a CT scanner was available directly in the resuscitation room. The study results indicate that 76.6% (*n* = 36) of the hospitals reported having a radiologist available in the ED 24/7. Additionally, 48.9% (*n* = 23) reported having a radiologist in the resuscitation room. Teleradiology access was available 24/7 in 68.9% (*n* = 31) of the hospitals. Furthermore, 93% (*n* = 44) of the responding hospitals reported having an MTRA available 24/7 during treatment in the resuscitation room.

**Fig. 2:**
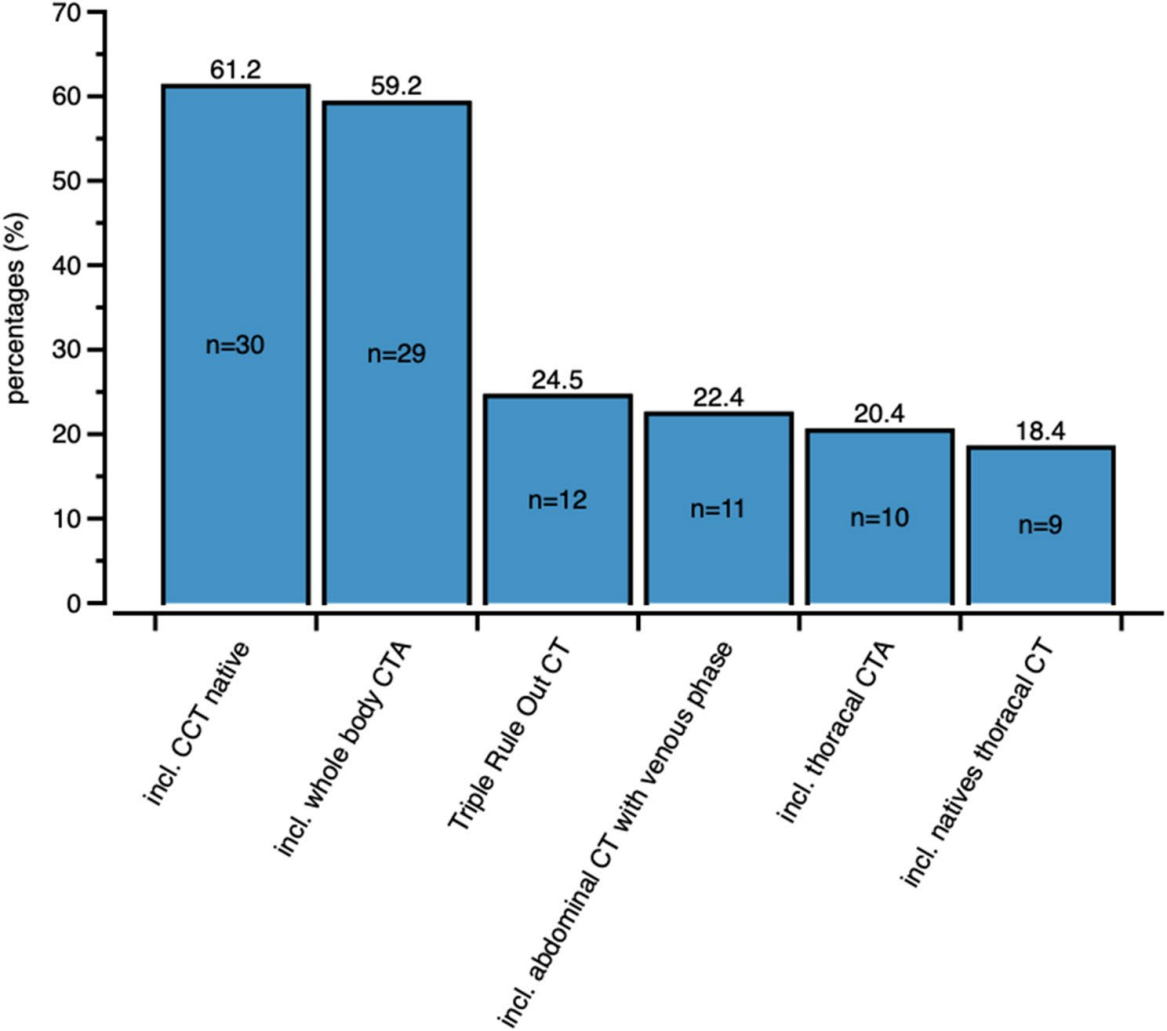
Types of CT imaging methods used in hospitals and post-RCT protocols (*n* = 49). At least one hospital can apply one method

The question regarding cross-sectional imaging, including a postresuscitation CT protocol, was answered using a multiple-choice format and describing the status quo of CT imaging. In 61.2% (*n* = 30 out of *n* = 49) of thehospitals, a standard non-contrast CT of the head was performed; in another 59.2% (*n* = 29) of the hospitals, a standard whole-body CT was used from the head to the pelvis; in 24.5% (*n* = 12) of the hospitals, a triple-rule-out CT was used; and in 22.4% (*n* = 11) a standard CT of the abdomen was used in the portal venous phase; in 20.4% (*n* = 10), a standard CT of the thorax was used; and in 18.4% (*n* = 9) of the hospitals, a standard CT of the thorax without contrast agent was used (Fig. 2).

### Other imaging findings

**Fig. 3:**
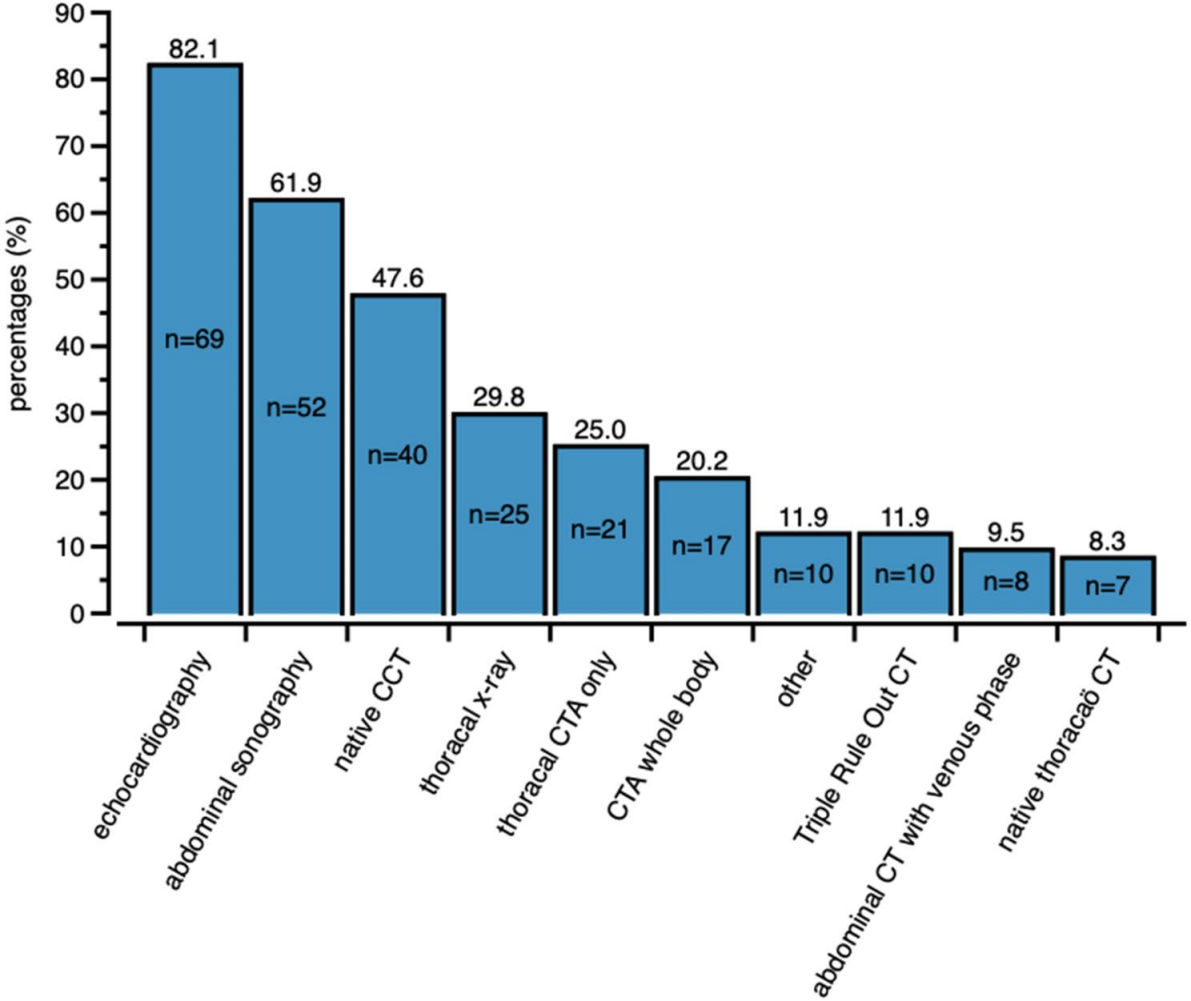
Standard diagnostic methods in hospitals without (*n* = 93) postrCT protocols. More than one method can be applied by one hospital

In hospitals without a postresuscitation CT protocol, further distinct diagnostic modalities were echocardiography (82.1%, *n* = 69), abdominal sonography (61.9%, *n* = 52), standard CT of the head (47.6%, *n* = 40), standard chest X-ray (29.8%, *n* = 25), standard CT angiography of the chest (25.0%, *n* = 21), and CT angiography from the head to the pelvis (20.0%, *n* = 17) (Fig. 3). A sonography protocol was used in 70.4% (*n* = 95) of the hospitals, with extended focused assessment with sonography for trauma (eFAST) (48.36%, *n* = 59) and “Rapid Ultrasound in Shock” RUSH (31.2%, *n* = 38) being the most common. A total of 52.2% (*n* = 71) and 30.2% (*n* = 41) of the sonography were performed by emergency physicians and internists, respectively.

In 89.7% (*n* = 122) of the hospitals, the same sonography protocol was used for both stable and unstable patients.

## Comparison of CAC-certified and non-CAC-certified hospitals

A total of 44 (40.7%) of the 108 German CAC-certified hospitals responded to the survey at the time. As shown in Table 1, a comparison of the mean number of patients treated per year between CAC-certified hospitals (*n* = 44) and noncertified hospitals (*n* = 110) showed that although the mean number of ED patients treated in CAC-certified hospitals (mean: 27.0k) was greater than that in noncertified hospitals (mean: 23.9k), the difference was not statistically significant (*p* = 0.075). A postresuscitation protocol was significantly more common in the CAC-certified hospitals (92.1%, *n* = 35) than in the non-CAC-certified hospitals (49.0%, *n* = 51, *p* < 0.001). Similarly, the use of a postresuscitation CT protocol was significantly more common in the CAC-certified hospitals (63.2%, *n* = 24) than in the non-CAC-certified hospitals (22.5%, *n* = 23, *p* < 0.001).

The analysis showed that CAC-certified hospitals (69,0%, *n* = 29) performed significantly more CT scans within six hours after successful interventions in the CCL (46,2%, *n* = 50, *p* = 0.02014) and following without intervention in the CCL examinations [81.4% (*n* = 35) vs. 60.0% (*n* = 42), *p* = 0.02105]. However, after Bonferroni correction, this difference was no longer statistically significant. Furthermore, CAC-certified hospitals were found to have logistical advantages over non-CAC-certified hospitals, as indicated by the greater prevalence of CT rooms and CCLs in close proximity. Specifically, the CT room was located directly in the ED in 20.9% (*n* = 9/43) of the CAC-certified hospitals, while only 5.5% (*n* = 6/110) of the non-CAC-certified hospitals had this resource (*p* = 0.008184). Similarly, the CCL was available in close proximity (less than 50 m) in 46.5% (*n* = 20/43) of the CAC-certified hospitals compared with 30.7% (*n* = 31/101) of the non-CAC-certified hospitals, with a nominally significant p value of 0.03782.

**Table 1:**
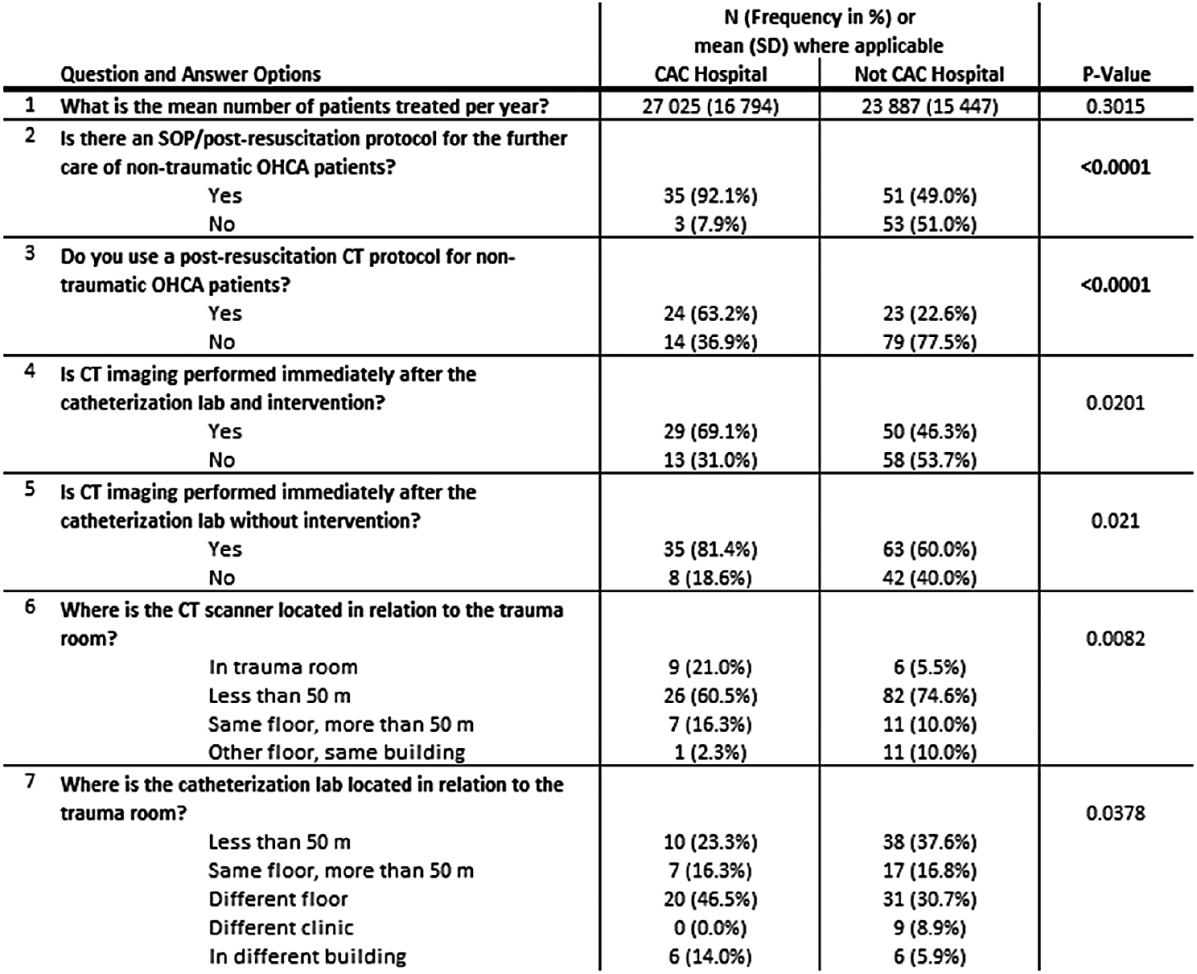
Comparison of hospitalized CAC-certified (CAC Hospital) and non-CAC-certified (not CAC Hospital) hospitals based on their responses to survey questions. Since the number of CAC-certified hospitals participating in the survey was lower than the number of non-CAC-certified hospitals, the following statistical tests were used: a Welsh two sample t test was used for comparing means, and Pearson’s chi-square test was used with Yates’ continuity correction for comparing frequencies. Statistically significant values after bonferroni correction are highlighted in bold

## Discussion

This anonymized cross-sectional study, conducted using an online survey and a standardized questionnaire, included 182 hospitals with 121 hospitals answering all questions, 44 of whom were German CAC-certified hospitals. Although the number of participants in this German ED survey was lower compared to other status quo assessments, it remains a valuable baseline study [15]. For the first time, this survey provides an overview of Germany’s emergency department landscape, focusing on structure, processes, and the use of post-resuscitation CT scan protocols in OHCA patients. While online surveys offer several advantages, low response rates are a common challenge, and their impact on result validity remains uncertain [16]. However, previous research indicates that a lower response rate does not necessarily introduce bias, particularly when key subgroups—such as CAC-certified hospitals in this survey—are well represented [16]. The study revealed a disparity in the availability and use of CT in EDs: while only 9.6% of EDs had CT capabilities directly in the resuscitation room, 70.1% had a CT scanner in close proximity (≤ 50 m). Specific postresuscitation CT protocols are available for only 30.0% of participating EDs.

These findings suggest that despite the presence of radiologists, MTRAs, or teleradiology agents, improvements can be achieved only through uniform standardization, highlighting the need for wider implementation of postresuscitation CT protocols. Adoption and adherence to standardized protocols could help to improve the consistency and quality of care and increase diagnostic accuracy.

The survey showed that in hospitals without a specific postresuscitation CT protocol, other distinct methods, particularly echocardiography (82.1%) and abdominal sonography (61.9%), were used. Although these methods are rapidly deployable and well established in clinical practice, they rely heavily on the experience of the examiner and may provide limited organ-specific information. In contrast, CT imaging, particularly in the postresuscitation setting, has been shown to be sensitive to complications resulting from resuscitation, including underlying causes of cardiac arrest [12, 17]. These findings suggest that there may be valuable opportunities to further integrate CT into postresuscitation protocols. Additionally, the establishment of transesophageal echocardiography (TEE) during ongoing resuscitation efforts could also play an important role, given its potential relevance in these critical situations, but this question was not addressed in this survey, as this procedure is not yet standard in the clinical practice in the German EDs [18].

One of the most commonly used CT methods is head CT, which is performed in only 47.6% of hospitals, despite several studies demonstrating the high diagnostic value of immediate head CT. For example, a study by Inamasu et al. (2009) showed that head CTs performed within 40 min on patients with witnessed nontraumatic OHCA who subsequently achieved hemodynamic stability were diagnosed with subarachnoid hemorrhage (SAH) in 16.2% of the patients in Japan/Asia [19]. Similarly, the diagnostic value of early head CT after OHCA has been confirmed in other studies, indicating that alternative methods may not uncover all life-threatening pathologies [20, 21].

Moreover, whole-body CT provides a more comprehensive diagnostic approach that goes beyond the assessment of internal organs and offers a wider range of diagnostic capabilities. The increasing adoption of eCPR has further underscored the critical importance of prompt CT diagnostics. Continuous anticoagulation therapy during eCPR presents significant challenges in the management of bleeding complications due to preexisting pathological hemostasis, complicating clinical practice. For example, a study by Yang et al. (2020) on eCPR therapy after OHCA found clinically relevant CT findings in 77.4% of patients [22]. Furthermore, while early and routine standardized whole-body post-resuscitation CT is crucial for guiding intensive care decisions—particularly regarding anticoagulation therapy, bleeding risk management, and prognostic assessments, including end-of-life decisions in the ICU—its benefits must be weighed against potential risks. However, in critically ill patients, the focus is on survival and neurological recovery, making concerns about contrast-induced nephropathy (CIN) less relevant in clinical decision-making. The concern about CIN should not overshadow the diagnostic value of contrast-enhanced imaging in OHCA patients. This principle is also reflected in studies on less critically ill patient groups, such as those with non-traumatic acute abdomen, where the benefits of contrast-enhanced CT were deemed to outweigh the risks of CIN [23]. Given that OHCA patients face significantly higher mortality and morbidity risks, this argument becomes even more relevant in their context.

Although CIN has traditionally been considered a potential complication, its clinical relevance in this context is limited. OHCA patients are already at high risk for acute kidney injury (AKI) due to prolonged hypoperfusion and pre-existing comorbidities, with multiple factors contributing to renal dysfunction beyond contrast media alone. Moreover, evidence suggests that early (< 24 h) contrast administration is not associated with an increased risk of AKI in OHCA survivors, even after adjusting for confounders [24].

Standardized protocols should therefore focus on optimizing imaging strategies to guide immediate and evidence-based treatment decisions rather than being overly restrictive due to nephrotoxicity concerns. While general renal protective strategies, such as hydration, remain prudent, they should not delay or limit the use of contrast-enhanced imaging when clinically indicated.

## Differences between CAC and Non-CAC certified hospitals

There was a significant difference between CAC-certified and noncertified hospitals. The survey results indicate that CAC-certified hospitals are more likely to use a postresuscitation protocol and a specific postrCT protocol. Furthermore, these hospitals have logistical advantages, such as the proximity of CT scanners and CCLs to the ED (as indicated by a nominal significance of *p* = 0.03782). In CAC-certified hospitals, hardly any CT scanners were located in another building; however, in more than 20.9% of the hospitals, the CT scanner was located in the resuscitation room. This makes it easier to carry out diagnostic and therapeutic procedures quickly. CAC-certified hospitals are significantly more likely to perform CT scans within six hours after patient admission, both before and after a CCL examination with or without intervention. These consequent use of protocols (including CT imaging) due to CAC certification standards maybe helpful for neurological outcomes in the OHCA patients. A recent retrospective observational study in 2023 from Voß et al. among three university hospitals about the impact of the CAC before and after certification on patient outcome in Germany showed that the overall survival remained similar but the likelihood of favourable neurological status at discharge was significantly higher after CAC accreditation [25].

In contrast to the German findings, the prospective multicenter randomized trial by Patterns et al. in the UK, the ‘ARREST Trial,’ found no significant difference in survival rates or neurological outcomes for OHCA patients without ST-elevation, whether they were referred to a cardiac arrest center or the geographically closest emergency department. However, this study has notable limitations, including the inclusion of a highly heterogeneous patient population, potential dilution effects due to non-cardiac causes of arrest, and the fact that even non-CAC hospitals in the trial had access to high-quality post-resuscitation care, potentially masking differences in outcomes [26].

Building on these observations, another study by Markus et al. (2024) emphasizes the need for a standardized approach, particularly highlighting the critical role of whole-body CT in the management of OHCA patients. Their findings corroborate our results, demonstrating that in CAC-certified hospitals, CT scans are routinely performed both following nonsignificant CCL findings and after reperfusion therapy. In their retrospective study conducted at a CAC-certified hospital (University Hospital of Marburg) between January 2018 and December 2022, 545 patients with nontraumatic OHCA were analyzed. Among these patients, 87.9% (*n* = 391) underwent early whole-body CT, and 72.4% (*n* = 322) received invasive coronary diagnostics. Due to the efficiently coordinated infrastructure that streamlined patient care, the additional time required for whole-body CT was limited to no more than 10 min. The study further revealed that in this patient cohort, the survival rate at hospital discharge was 39.8% (*n* = 217/545), reinforcing the significance of early-stage CT imaging and highlighting the importance of standardized protocols. This approach facilitates the prompt identification of underlying conditions leading to OHCA, as well as complications associated with CPR [27]. Considering these findings, the incorporation of postresuscitation CT into the care pathway for OHCA patients may offer significant clinical benefits.

However, its use must be carefully balanced against potential risks, including radiation exposure and contrast-induced complications, which could potentially lead to further harm and impact survival outcomes. While further research is necessary to definitively establish the benefits of increased standardization, current findings contrast with previous observational studies on cardiac arrest centers, which reported improved survival and/or neurological outcomes [3, 24–29].

### Logistical challenges and further research

The resuscitation room of the ED is essential for the initial diagnosis and stabilization of patients who have suffered from OHCA. When acute coronary syndrome is suspected or ST-elevation/ST-elevation myocardial infarction (STEMI) is verified, the CCL becomes a critical diagnostic pillar. Managing patients with suspected acute coronary syndrome presents specific challenges. Notably, EMSs frequently transport patients with STEMI-induced OHCA directly to the CCL, bypassing the ED, which is considered the optimal approach for ensuring timely and effective patient care. This approach helps to meet the 90-minute time frame from diagnosis to initiation of reperfusion therapy [30].

It is important to note that not all STEMI diagnoses are accurate. A small study by Compagnoni et al. (2021) revealed that 29.6% (16/54) of STEMI diagnoses were false positives due to low perfusion after OHCA without vessel occlusion during cardiac catheterization [31]. Another study by Salam et al. (2016) showed that out of 48.0% (*n* = 78) of patients with ECG findings indicating STEMI, 48.0% had STEMI, 21.0% had non-STEMI, and 31.0% had no myocardial infarction [32]. In a multicenter study by Baldi et al. (2020), statistically significant false-positive ECG changes were found to be particularly prominent in the first 8 min post-ROSC. Based on these findings, it would be prudent to utilize an ECG for STEMI diagnosis after 8 min or at least repeat the ECG to ensure the accuracy of the diagnosis [33]. These findings highlight the challenges of accurately diagnosing STEMI and emphasize the importance of comprehensive diagnostic approaches to avoid misinterpretation and ensure appropriate patient management and allocation. Several randomized controlled trials like the TOMAHAWK study have shown that immediate vs. delayed angiography strategy had no benefit for the patients after successful resuscitation without ST elevation [34–36].

In the management of out-of-hospital cardiac arrest (OHCA) patients, it is essential to establish a well-structured protocol that defines both the optimal timing for transport to the cardiac catheterization laboratory (CCL) and the appropriate sequencing of a head-to-pelvis CT scan in relation to the CCL examination. While potential risks associated with CT imaging—such as radiation exposure and contrast-related complications—must be carefully evaluated, recent evidence suggests that contrast administration within 24 h was not associated with an increased risk of acute kidney injury among survivors of sudden cardiac arrest [24]. This information should guide emergency department staff in making informed decisions while balancing the benefits and risks of imaging procedures.

Furthermore, subsequent steps should be determined based on whether the CCL findings are significant or nonsignificant. Emerging evidence suggests that even after a successful intervention in the CCL, a timely CT scan can be valuable in identifying complications resulting from CPR. This additional diagnostic step may therefore play a crucial role in optimizing patient care and guiding further treatment decision [27, 37].

Uniform standardization is desirable, as the prognosis of resuscitated patients strongly depends on the speed and quality of treatment and the cause of resuscitation. Another potential influencing factor is logistical disadvantages, such as the distance between the ED’s resuscitation room or CT room and the CCL. This has a negative impact on the time-critical diagnostic process. It would therefore be advisable to minimize these distances, as these three pillars are essential for adequate and timely diagnosis and treatment of this vulnerable patient group. Currently, there are no established definitions of the timing (immediately, within two hours, within six hours), extent (head, pelvis, thorax, whole body) or type (venous phase, arterial phase, non-contrast) of CT examination after OHCA. Furthermore, the results can serve as a basis for further clinical research and potentially for the establishment of standardized guidelines for CT imaging after nontraumatic OHCA in Germany.

## Limitations

This study surveyed emergency department across Germany. While almost all federal states were represented, the participation rate was relatively low, possibly due to the comprehensive nature of the questionnaire. Therefore this results may have a reduced interpretability and generalizability of its results.

Additionally, the voluntary nature of participation in the survey may lead to selection bias and potential data bias. Furthermore, not all participants answered all the questions completely, resulting in data gaps. Finally, the lack of standardized protocols makes it difficult to compare results and derive general recommendations.

In conclusion, the results of this status quo survey suggest general heterogeneity in imaging diagnostics and care of OHCA patients in German EDs.

## Electronic supplementary material

Below is the link to the electronic supplementary material.

Supplementary Material 1

## Data Availability

All data supporting the findings of this study are available within the manuscript and its Supplementary Information.

## Abbreviations

CAC: Cardiac arrest center
CCL: Cardiac catheterization laboratory
CT: Computed tomography
CT-PA: Computed tomography pulmonary angiography
DGINA: German society for interdisciplinary emergency and acute medicine
DIVI: German interdisciplinary association for intensive care and emergency medicine
DGIIN: German society for medical intensive care and emergency medicine
DGK: German society of cardiology
ED: Emergency department
EMS: Emergency medical services
eFAST: Extended focused assessment with sonography for Trauma
eCPR: Extracorporeal cardiopulmonary resuscitation
ICU: Intensive care unit
MTRA: Medical technical radiology assistant
OHCA: Out-of-hospital cardiac arrest
ROSC: Return of spontaneous circulation
RUSH: Rapid ultrasound in shock
SOP: Standard operating procedure
STEMI: ST-elevation myocardial infarction
TEE: Transesophageal echocardiography
