# Supplementary material for: CT imaging in post-resuscitation care of non-traumatic resuscitation room patients in German hospitals
Source: BMC Emerg Med. 2025 Apr 15;25:63. doi: 10.1186/s12873-025-01216-w (PMC12001513; doi:10.1186/s12873-025-01216-w)
Supplement: Supplementary file 1 — Supplementary Material 1 [file 12873_2025_1216_MOESM1_ESM.docx]

### CT Imaging in Post-Resuscitation Care of Non-Traumatic Resuscitation Room Patients in German-Speaking Hospitals

In hospitals, the approach following out-of-hospital cardiac arrest (OHCA) is very heterogeneous: the admission of patients after OHCA occurs in different locations within the hospital and follows different treatment protocols. This leads to advanced imaging diagnostics using computed tomography (CT) for determining causes and assessing consequences of non-cardiac causes and resuscitation outcomes at different examination phases and intervals.

This survey captures the diagnostics in post-resuscitation treatment in non-traumatological emergency rooms in German-speaking hospitals and is aimed at the medical management structure of emergency departments.

Please take 10 minutes to answer this online survey. The goal is to publish the results in anonymized form and, with your help, gain a better understanding of imaging during post-resuscitation treatment in German emergency departments, thereby improving emergency room care for critically ill patients.

We thank you for your cooperation!

On behalf of the working groups of the respective professional societies:

- Dr. Domagoj Schunk (DGINA)
- Prof. Dr. Bernhard Kumle (DGINA)
- Prof. Dr. Guido Michels (DGK)
- Prof. Dr. Hans-Jörg Busch (DGIIN)
- Prof. Dr. Uwe Jansens (DIVI)

Participation in the study is anonymized. Once you submit the anonymized questionnaire, it cannot be withdrawn later.

Do you agree to participate in the study?

Yes

No

### Your Hospital

Please provide the key information about your hospital below. Please ensure that only one questionnaire is completed per emergency department and that this questionnaire is not intended for pediatric emergency departments.

Do you represent a pediatric emergency department?

Yes (exit question)

No

Location of the hospital, please enter the federal state:

Please indicate the population size of the city where the hospital is located.

0 - 50,000 inhabitants

50,000 - 100,000 inhabitants

100,000 - 200,000 inhabitants

200,000 - 500,000 inhabitants

500,000 - 1 million inhabitants

more than 1 million inhabitants

Classification of the emergency department according to G-BA levels:

Basic emergency care

Advanced emergency care

Comprehensive emergency care

Classification of the hospital's level of care:

Basic care

Regular care

Specialized care

Maximum care

Supramaximal care - University clinic

Number of planned beds (excluding psychiatry and rehabilitation):

<200

201-499

500-1000

1000

### Demographics of the Respondents

Please indicate your age:

How many years of specialist professional experience do you have?

< 5

6-10

11-20

20

What specialist qualifications do you have? (Multiple selections possible)

Internal Medicine

Internal Medicine and Cardiology

Internal Medicine and Pulmonology

Internal Medicine and Nephrology

Anesthesiology

Trauma Surgery/Orthopedics

Neurology

Other (please specify):

What additional qualifications do you have? (Multiple selections possible)

Intensive Care Medicine

Emergency Medicine

Clinical Acute and Emergency Medicine

Other (please specify):

None

### Structure of Your Emergency Department

Do you have a central, interdisciplinary emergency department?

Yes

No

Do you have a specialized emergency outpatient clinic?

No

Yes

Please specify the specialty:

### Structure of Your Emergency Department

Please provide key information about your emergency department where patients are admitted after non-traumatological OHCA.

Please indicate the number of patients treated in your emergency department per year:

Please indicate the number of treatment places (excluding emergency rooms) in your emergency department:

Is there an observation unit attached to the emergency department (for monitoring potentially to moderately critically ill patients requiring acute inpatient diagnostics and therapy)?

No

Yes

Please specify the number of beds:

Is there an admission unit attached to the emergency department (for clarifying and treating non-critically ill patients who need further standard inpatient clarification and care)?

No

Yes

Please specify the number of beds:

Is the intensive care unit (ICU) organizationally linked to the emergency department (same medical management)?

No

Yes

Please specify the number of beds:

### Diagnostic Capabilities in Your Hospital

Please provide key figures on the CT scanners, emergency rooms, and cardiac catheter laboratories available in your hospital:

Please indicate the number of emergency rooms in your emergency department:

Do you have an emergency room exclusively for traumatological patients?

No

Yes

Please specify the number:

Do you have an emergency room exclusively for non-traumatological patients?

No

Yes

Please specify the number:

Do you have emergency rooms in the ICU?

No

Yes

Please specify the number:

Please indicate the number of CT scanners in your hospital:

Where is the CT located in relation to the emergency room?

The CT is located in the emergency room

The CT is located in close proximity (< 50 m) to the emergency room

The CT is not in close proximity (> 50 m) (same level)

The CT is in the same building but on a different floor

The CT is in a different building (external transport required)

Is a radiologist available in the hospital 24/7?

Yes

No

Is a radiologist available in the emergency room 24/7?

Yes

No

Is teleradiology available 24/7?

Yes

No

Are a radiologist and teleradiology available 24/7?

Yes

No

Is an MTRA available in the emergency room 24/7?

Yes

No

Do you have a certified Chest Pain Unit in the hospital?

Yes

No

### Diagnostic Capabilities in Your Emergency Department

Where is the Chest Pain Unit located?

Integrated into a normal ward

Integrated into the emergency department

Integrated into an admission unit

Integrated into an observation unit

Integrated into an intermediate care unit (IMC)

Integrated into an ICU

The CPU is a standalone unit/station

Other, please specify:

Please indicate the number of cardiac catheter laboratories in the hospital:

Where is the cardiac catheter laboratory located in relation to the emergency room?

The cardiac catheter laboratory is in close proximity (< 50 m)

The cardiac catheter laboratory is not in close proximity (> 50 m) (same level)

The cardiac catheter laboratory is on a different floor

The cardiac catheter laboratory is in a different building

The cardiac catheter laboratory is in an external clinic (e.g., outsourced heart center)

Is a post-resuscitation CT performed immediately (within 6 hours after admission) following primary cardiac arrest (OHCA) and after intervention for non-traumatological patients, such as a heart attack?

Yes

No

Is a post-resuscitation CT performed within 6 hours after admission following primary cardiac catheterization without intervention and without indication of a primary rhythmogenic or cardiogenic event for non-traumatological OHCA patients?

Yes

No

### Cardiac Arrest Center (CAC)

Do you also treat traumatological resuscitation patients?

Yes

No

Are you certified as a CAC (DGK/GRC)?

Yes

No

Is your clinic planning certification?

Yes

No

What is the composition of the admission team in terms of doctors? (Multiple selections possible)

Internist

Cardiologist

Anesthetist

Intensive Care Physician

Acute/Emergency Physician

Radiologist

Other (please specify):

What is the composition of the nursing team? (Multiple selections possible)

Intensive Care Nursing

Anesthesia Nursing

Emergency Department Nursing

Cardiac Catheter Laboratory Nursing

Other (please specify):

Who leads the admitting team?

ICU Physician

Anesthetist

Emergency Department Physician

Cardiologist

Other (please specify):

Do you have a CART (Cardiac Arrest Resuscitation Team) in the emergency department?

Yes

No

Is the emergency department specialist part of the CART?

Yes

No

Is the emergency department nurse part of the CART?

Yes

No

Who leads the CART team?

ICU Physician

Anesthetist

Emergency Department Physician

Cardiologist

Other (please specify):

If the CART is not in the emergency department, where is the CAR team located?

ICU

Cardiac Catheter Laboratory

Radiology

Other (please specify):

What is the medical composition of the CAR team? (Multiple selections possible)

Internist

Cardiologist

Anesthetist

Intensive Care Physician

Acute/Emergency Physician

Radiologist

Other (please specify):

What is the nursing composition of the CAR team? (Multiple selections possible)

Intensive Care Nursing

Anesthesia Nursing

Emergency Department Nursing

Cardiac Catheter Laboratory Nursing

Other (please specify):

### Concepts for the Treatment of Non-Traumatological OHCA Patients

Do you have an SOP/post-resuscitation protocol for the continued care of non-traumatological OHCA patients?

Yes

No

Does the handover location change depending on the suspected etiology of the non-traumatological OHCA patient?

Yes

No

For which cardiac causes of OHCA is the handover performed in the cardiac catheter laboratory? (Multiple selections possible)

Acute coronary syndrome (e.g., STEMI)

History suggesting a cardiac event (e.g., patient clutched their chest, sudden cardiac death during sports, or known CAD/heart failure patient)

Ventricular arrhythmias (ventricular tachycardia, ventricular fibrillation)

Left bundle branch block/right bundle branch block

Ongoing resuscitation with suspected cardiac origin

Cardiogenic shock patients with indication for eCPR (ECLS installation in the cardiac catheter laboratory)

Other (please specify)

Where is the handover performed for suspected non-cardiac causes after an OHCA?

Handover in the emergency department

Handover in the ICU

Handover in the cardiac catheter laboratory

Other (please specify)

Where are your non-traumatological OHCA patients primarily cared for?

Non-traumatic OHCA patients are primarily cared for in the emergency room in the emergency department

Non-traumatic OHCA patients are primarily cared for in the emergency room in the ICU

Non-traumatic OHCA patients are primarily cared for in the cardiac catheter laboratory

Primary care location changes based on the reported condition

Other (please specify):

How many patients are admitted to your emergency room per year following non-traumatological OHCA? Approx.:

### Computed Tomography Protocol for Non-Traumatological OHCA in the Emergency Department

Do you use a post-resuscitation CT protocol for non-traumatological OHCA patients?

Yes

No

What exactly does the post-resuscitation CT protocol for non-traumatological OHCA patients include? (Multiple selections possible)

Includes a standard native CT of the head

Includes standard CT angiography from head to groin

Includes only standard CT angiography of the thorax

Includes standard native CT of the thorax

Includes standard CT of the abdomen in the portal venous phase

Triple-Rule Out CT

Is a standard chest X-ray performed before the CT?

Yes

No

If you do not have a post-resuscitation CT protocol, please specify which examinations are routinely performed for non-traumatological OHCA patients? (Multiple selections possible)

Standard native CT of the head

Standard CT angiography from head to groin

Standard CT angiography of the thorax only

Standard native CT of the thorax

Standard CT of the abdomen in the portal venous phase

Triple-Rule Out CT

Standard chest X-ray

Echocardiography

Abdominal ultrasound

Other (please specify):

### Ultrasound Protocol for Non-Traumatological OHCA Patients

Do you use an ultrasound protocol for non-traumatological OHCA patients?

Yes

No

Which of the following ultrasound protocols do you use for non-traumatological OHCA patients?

eFAST

FEEL

FATE

RUSH

Other (please specify)

Who usually performs the ultrasound in the emergency room?

Radiologist

Internist

Cardiologist

Acute/Emergency Physician

Anesthetist

Intensive Care Physician

Other specialties:

Do you have different ultrasound protocols for stable/unstable (non-traumatological OHCA) patients in your emergency department?

Yes

No

Which ultrasound protocol do you use for non-traumatological, stable patients after OHCA?

Which ultrasound protocol do you use for unstable, non-traumatological OHCA patients?

### Standard Diagnostics after Non-Traumatological OHCA in the Emergency Department

Please specify which standard diagnostics you perform after non-traumatological OHCA in the emergency department? (Multiple selections possible)

12-lead ECG

Chest X-ray

CT imaging

Echocardiography

Abdominal ultrasound

Venous blood gas analysis (BGA)

Arterial blood gas analysis (BGA)

Laboratory diagnostics

Temperature measurement

Continuous temperature measurement

Other (please specify):

Please indicate the location of temperature measurement: (Multiple selections possible)

Tympanic

Nasal

Oral

Axillary

Rectal

Vesical

Other (please specify):

### Therapy and Diagnostics in the Emergency Room

What measures are taken in your emergency room after non-traumatological OHCA? (Multiple selections possible)

Temperature management (TTM)

Control of hypoxia, hypercapnia, oxygenation, and ventilation parameters

Use of specific TTM tools

Treatment of hyper- or hypoglycemia to achieve normoglycemia

Muscle relaxation as standard in OHCA

Blood pressure management (e.g., catecholamine therapy)

Use of mechanical resuscitation aids

Insertion of an arterial catheter

Insertion of a central venous catheter

Insertion of an ECLS (eCPR)

Which method do you predominantly use for cooling? (Multiple selections possible)

Thermogard

Arctic Sun

RhinoChill

Cooled infusions

Cool packs

Ice cubes

Other (please specify)

### Intensive Care Unit (ICU) Structure

How many ICUs for adult patients do you have? (Please specify the number.)

Please indicate the number of ICU beds for adult patients in your hospital:

On which ICUs are non-traumatological post-resuscitation patients primarily cared for?

Internal Medicine

Cardiology

Cardiac Surgery

Pulmonology

Anesthesiology

Neurosurgery

Neurology

Interdisciplinary

Other (please specify):

Please indicate where the ICU is located:

In close proximity to the emergency department (< 50 m)

Not in close proximity to the emergency department (> 50 m) (same level)

In another building (external transport necessary)

In the same building, but on a different floor

### Interface with Emergency Services

Is your emergency department actively involved in the emergency medical service?

Yes

No

Do you conduct joint resuscitation training with the emergency services?

Yes

No

Is your emergency department actively involved in the training of emergency services (e.g., as an instructor)?

Yes

No

Exclusion Criterion: This questionnaire is not intended for pediatric emergency departments.
